# Characterization of Transcription Factors That Regulate the Type IV Secretion System and Riboflavin Biosynthesis in *Wolbachia* of *Brugia malayi*

**DOI:** 10.1371/journal.pone.0051597

**Published:** 2012-12-10

**Authors:** Zhiru Li, Clotilde K. S. Carlow

**Affiliations:** New England Biolabs, Division of Parasitology, Ipswich, Massachusetts, United States of America; Biomedical Research Institute, United States of America

## Abstract

The human filarial parasite *Brugia malayi* harbors an endosymbiotic bacterium *Wolbachia* (*w*Bm) that is required for parasite survival. Consequently, targeting *w*Bm is a promising approach for anti-filarial drug development. The Type IV secretion system (T4SS) plays an important role in bacteria-host interactions and is under stringent regulation by transcription factors. In *w*Bm, most T4SS genes are contained in two operons. We show the *w*Bm is active since the essential assembly factor *virB8-1*, is transcribed in adult worms and larval stages, and VirB8-1 is present in parasite lysates. We also identify two transcription factors (*w*BmxR1 and *w*BmxR2) that bind to the promoter region of several genes of the T4SS. Gel shift assays show binding of *w*BmxR1 to regions upstream of the *virB9-2* and *wBmxR2* genes, whereas *w*BmxR2 binds to *virB4-2* and *wBmxR1* promoter regions. Interestingly, both transcription factors bind to the promoter of the *ribA* gene that precedes *virB8-1*, the first gene in operon 1 of the *w*Bm T4SS. RT-PCR reveals *ribA* and *virB8-1* genes are co-transcribed as one operon, indicating the *ribA* gene and T4SS operon 1 are co-regulated by both *w*BmxR1 and *w*BmxR2. *RibA* encodes a bi-functional enzyme that catalyzes two essential steps in riboflavin (Vitamin B2) biosynthesis. Importantly, the riboflavin pathway is absent in *B. malayi*. We demonstrate the pathway is functional in *w*Bm, and observe vitamin B2 supplementation partially rescues filarial parasites treated with doxycycline, indicating *Wolbachia* may supply the essential vitamin to its worm host. This is the first characterization of a transcription factor(s) from *w*Bm and first report of co-regulation of genes of the T4SS and riboflavin biosynthesis pathway. In addition, our results demonstrate a requirement of vitamin B2 for worm health and fertility, and imply a nutritional role of the symbiont for the filarial parasite host.

## Introduction

Lymphatic filariasis and onchocerciasis are neglected tropical diseases caused by filarial nematode parasites. *Brugia malayi* and *Wuchereria bancrofti* reside in the lymphatics resulting in lymphedema, hydrocele and elephantiasis. Infection with *Onchocerca volvulus* leads to dermatitis and blindness. Collectively, these severely debilitating diseases afflict 150 million people in the tropics and threaten the health of over one billion. The parasites contain an α-proteobacterial endosymbiont belonging to the genus *Wolbachia*, where the bacterium is an obligate mutualist required for development, reproduction and survival of the parasite [1], [2], [3], [4]. There has been considerable interest in filarial *Wolbachia* since antibiotic-mediated clearance of *Wolbachia* from the parasite correlates with a block in embryogenesis, worm death and improvement in disease symptoms [3], [5], [6], [7], [8].

*Wolbachia* is also highly prevalent in insects with an estimated 20–80% of arthropod species infected [9], [10], [11], [12], including those that transmit human filarial parasites [13]. *Wolbachia*-host interactions in insects are complex and infection can result in a number of developmental and reproductive abnormalities [14], [15], [16]. Currently little is known about the molecular and/or biochemical basis of the interactions that exist between *Wolbachia* and its insect or nematode host. This is of great interest given the major effects on host biology and the possibility of exploiting the symbiotic relationship as a target for new drug discovery.

Many symbiotic and pathogenic intracellular bacteria use a type IV secretion system (T4SS) for successful infection, proliferation and persistence within hosts [17], [18], [19]. The T4SS of gram-negative bacteria is a trans-membrane channel composed of several conserved proteins that can transport effector molecules across the membrane into the cytoplasm or nucleus of eukaryotic cells in an ATP-dependent manner [20]. The system is present and highly conserved in insect *Wolbachia* [21], [22], [23], , which may implicate its importance in the host-symbiont interaction and survival of bacteria within host cells. Genome analysis predicts the presence of a T4SS in the *Wolbachia* present in *B. malayi* (*w*Bm), suggesting that the system is also likely active in filarial *Wolbachia* [24]. Previous studies in other bacteria have shown that the T4SS is not constitutively expressed but tightly regulated by transcription factors [25], [26], [27], [28]. In the intracellular rickettsial pathogen *Ehrlichia chaffeensis*, a transcription factor EcxR has been identified which binds to the promoter regions upstream of *virBD* genes of the T4SS, as well as its own promoter, and positively regulates their expression in a developmental stage-specific manner [29].

In the present study, we demonstrate *w*Bm has an active T4SS since the essential assembly factor *virB8-1*, is transcribed in adult worms and larval stages, and VirB8-1 is present in parasite lysates. We also identify two transcription factors that regulate T4SS gene expression. Interestingly both transcription factors also bind to the promoter of the *ribA* gene located upstream of *virB8-1*, the first gene in operon 1 of the *w*Bm T4SS. We show *ribA* and *virB8-1* genes are co-transcribed as one operon, indicating the *ribA* gene and T4SS operon 1 are co-regulated. *RibA* encodes a bifunctional enzyme that catalyzes two essential steps in riboflavin (vitamin B2) biosynthesis. While the riboflavin pathway is absent from *B. malayi*, we demonstrate the pathway is expressed in *w*Bm. We find vitamin B2 supplementation partially rescues parasites treated with doxycycline, indicating *Wolbachia* may supply the essential vitamin to its worm host.

## Materials and Methods

### Identification of orthologs of EcxR in *w*Bm

To identify homolog(s) of *Ehrlichia* EcxR, protein sequence ECH_0795 (YP_507593) was used to query the genome of the endosymbiotic bacteria *Wolbachia* (*w*Bm) of *B. malayi* and *Wolbachia* of *Drosophila melanogaster* (*w*Mel). Protein alignment was performed using the TCOFFEE ClustalW alignment software (http://tcoffee.vital-it.ch/cgi-bin/Tcoffee/tcoffee_cgi/index.cgi) and displayed using BOXSHADE (www.ch.embnet.org/software/BOX_form.html). A phylogenetic tree was generated using ClustalW (http://align.genome.jp/sit-bin/clustalw). A protein blast search was performed to identify putative conserved domains belonging to the HXT_XRE superfamily of cl15761 that includes DNA binding proteins belonging to the xenobiotic response element family of transcriptional regulators. Protein structure prediction was performed using the PHYRE program [30]. Helix-turn-helix structure analysis was based on the structure of 2o38_A, a putative Xre family transcriptional regulator from the alpha-proteobacterium *Rhodopseudomonas palustris CGA009*. Sequence identity values between two sequences were generated using BlastP.

**Table 1:** Primers used for RT-PCR.

Gene	Primer Direction	Sequence 5′ to 3′	Amplicon size (bp)
*wBmxR1*	Forward	AACACTTGGACTCAAGCTCATGCC	120
	Reverse	CTTGAGAAACCCTAACAGTCGTTCT	
*wBmxR2*	Forward	GCAGCAATTGCCCTAAAGATCGAC	86
	Reverse	AGCAAGTATTCTAAAGAGAACCCTTCAA	
*ribA-virB8-1 intergene*	Forward	ACCTGATGCAAGATGGTAGAGGCA	600
	Reverse	ACGGTTCAATAGTGCTGCTAGTGC	
*ribA*	Forward	ATCTATTGGTTGCTGCTGCCGAGA	140
	Reverse	GCGTTTGCTACTATGCTCCACGTT	
*ribD*	Forward	AACCCTGCTGTCGGGTGTATCATT	122
	Reverse	TGGGTTGAACCTTTAGCGCTTTGC	
*ribB*	Forward	GCACGTAATGGTCATACTGAGGCAAG	112
	Reverse	GCAAGCGCATCATAGAACCGTCAT	
*ribE*	Forward	TGGGTGTGTAATTCGTGGTGAGAC	120
	Reverse	GCAGTGATTACACCCATACCAAGAGG	
*ribC*	Forward	GCTTGCTCTGGCGTGTGTTTAACT	100
	Reverse	ACGCACCCAGGTTAGAAACCTTCA	

### Quantitative RT-PCR to determine gene expression in various developmental stages of *B. malayi*

Total *B. malayi* RNA supplied by the Filariasis Research Resource Center (FR3) was treated with RNase-free Dnase (New England Biolabs, NEB) and purified using the RNeasy Kit from Qiagen. cDNA was obtained using random primers and M-MuLV reverse transcriptase (RT+) (NEB). Reactions containing no reverse transcriptase were included to detect potential DNA contamination of the RNA sample. Forward and reverse primers listed in Table 1 were used to amplify the desired sequences. *Wolbachia* 16S rRNA was used for bacterial total RNA quantification. Quantitative PCR was performed using the DyNAmo™ HS SYBR® Green qPCR Kit (Thermo Fisher) and a *CFX-96* Real Time PCR instrument (Bio-rad). To determine temporal gene expression in bacteria, quantification of the 16S rRNA is often used as a reference [31], therefore gene expression levels were calculated relative to 16S rRNA as previously described [32]. To detect expression of the intergenic region between *ribA* and *virB8*, PCR products were loaded onto a 1.2% agarose gel and stained with ethidium bromide.

### Cloning, expression and purification of recombinant proteins

Living *B. malayi* adult female worms were purchased from TRS Laboratories, Athens GA. Genomic DNA was isolated following the protocols developed by Dr. Steven A. Williams (http://www.filariasiscenter.org/molecular-resources/protocols).

**Table 2:** Primers used to construct expression plasmids.

Protein	Primer Direction	Sequence 5′ to 3′	Size (bp)
*w*BmxR1	Forward	gagaCATATGGAAATAATATCATTAAACAATAACTCGTGC	297
	Reverse	gagaCTCGAGTGGAGATCCAACTTCACGTTTAACC	
*w*BmxR2	Forward	ggaaCATATGCAAAATTCACAATTAAATTATCCTATAAC	279
	Reverse	gagaCTCGAGTTGTATGTTATGCTGAGAATTATGTTTTATTTTC	
VirB8-1_His6	Forward	ttttCATATGCCGTTCGTTATAGAAATTGAAAAAAAATCAG	684
	Reverse	ttttCTCGAGTACATATTCATCATCTACCCTATAAGAAGTAAC	
VirB8-1	Forward	ttttCATATGCCGTTCGTTATAGAAATTGAAAAAAAATCAG	681
	Reverse	ttttCTCGAGCTATACATATTCATCATCTACCCTATAAGAAGT	
ΔTMVirB8-1_His6	Forward	ttttCTCGAGCTATACATATTCATCATCTACCCTATAAGAAGT	492
	Reverse	ttttCTCGAGTACATATTCATCATCTACCCTATAAGAAGTAAC	

Restriction enzyme sites are underlined.

Additional nucleotides added to facilitate restriction enzyme digestion are shown in lower case.

Genes were amplified using genomic DNA isolated from *B. malayi*. Primers (Table 2) were synthesized according to the various gene sequences. Genes were cloned into the corresponding restriction enzyme sites of pET21a. The accuracy of the inserts was verified by sequencing. Plasmids were transformed into T7 express *E. coli* strain C2526 (NEB) for protein expression. For wBmxR2, optimum conditions for production of soluble recombinant proteins involved co-transformation of the pRIL plasmid (Agilent) together with *wBmxR2*-pET21a plasmids. Cultures were grown at 37°C till the OD_600_ reached 0.6, followed by induction with 0.1 mM IPTG overnight at 16°C. The cells expressing the recombinant proteins were suspended in lysis buffer (20 mM NaPO_4_, 500 mM NaCl, 10 mM imidazole, pH 7.4) plus 1 mg/mL lysozyme and protease inhibitor cocktail (Roche) and incubated on ice for 30 min, followed by sonication. The lysate was then cleared by centrifugation at 21,000 g, for 30 min at 4°C. The C terminus His_6_-tagged proteins were purified on a 5 ml HisTrap HP column (GE Healthcare) using an AKTA FPLC following manufacturer's instructions. After application of the sample, the column was washed with 5 column volumes of buffer A (20 mM NaPO_4_, 200 mM NaCl, 10 mM imidazole, pH 7.4) followed by 10 column volumes of 84% buffer A:16% buffer B (20 mM NaPO_4_, 500 mM NaCl, 400 mM imidazole, pH 7.4). Protein was then eluted using a linear gradient (8–100%) of buffer B equivalent to 40–400 mM imidazole. Fractions containing targeted protein were pooled, dialyzed against dialysis buffer (40 mM Tris-HCl, 200 mM NaCl and 50% glycerol, pH 7.5) and stored at −20°C prior to use. Purity of the proteins was evaluated by SDS-PAGE and the protein concentration was determined using the Bradford assay. The apparent molecular weights from SDS-PAGE, 12kDa for each protein were consistent with the predicted molecular size of *w*BmxR1 and *w*BmxR2 with a C-terminal His-tag.

### Western Blotting

Rabbit polyclonal anti-VirB8-1 antisera were purchased from Covance Inc., Cell lysates were diluted in 2× Laemmli sample buffer (Sigma Aldrich) and incubated at 96°C for 10 min. 20 μl of samples were subjected to SDS-PAGE using 4–20% Tris-HCl gels (Life Techology) and protein bands were transferred onto a 0.45 μm PVDF membrane using Trans-Blot SD semi-dry transfer cell (Bio-RAD). After blocking for 2 h in 4% milk diluted in Tris-buffered saline (TBS) containing 0.1% Tween, membranes were incubated overnight at 4°C in affinity-purified anti-VirB8-1 antibody diluted 1∶5,000 in blocking buffer. Membranes were washed at least four times in TBS with 0.1% Tween, and then incubated for one hour at room temperature in a 1∶2,000 dilution (TBS with 0.1% Tween) of goat-anti-rabbit-HRP antibody (Cell Signaling Technology). Following additional washing in TBS with 0.1% Tween, the blots were developed using the LumiGLO® reagent (Cell Signaling Technology).

**Table 3:** Primers used to amplify promoter regions of various genes.

*Gene*	Primer Direction	Sequence 5′ to 3′	Amplicon size (bp)
*ribA*	forward	TGTCTGGAATAACCTGAGACCACG	469
	reverse	GCCTAATTTCGCTGATGGCCCTTT	
*virB8-1*	forward	CAAAGAAAATATAACCTTGATACTGTGG	447
	reverse	CTGAGCAATAATTGTGCTGTAGCG	
*virB3*	forward	TCTGTTTGTATGCTGCCTGTGGAC	243
	reverse	AAGTACCAATATGCGGCAAACCCG	
*virB8-2*	forward	CGTTTTAGGATGAATATAAGTTTGCGG	466
	reverse	CTTGTCTTCCATAATATAAGGCCCTAGA	
*virB9-2*	forward	GAAACAGATAGAACACACCAAATCGAG	377
	reverse	CAGTACAACAAGCAGTAAACTAATCATG	
*virB4-2*	forward	TTCGGTGAGCGATGAGTTAAAG	357
	reverse	TGTTGGTGTTTTTACCTGCATACA	
*sodA*	forward	TTTACTCCTACGAGCATGCCCTTA	398
	reverse	AAGCTGTTTTATCATATGGTAACTCAGG	
*wBmxR1*	forward	CAGCGTCACGCAATGGAATAACAC	377
	reverse	CACTGCACGAGTTATTGTTTAATGA	
*wBmxR2*	forward	ATTTACCGTTTGCCTTGCGT	389
	reverse	TGCATGATAACAACTTCCTTATGA	

### Electrophoretic mobility shift assay (EMSA)

Promoter regions of 9 different genes were amplified by PCR using *B. malayi* total DNA and Phusion Hot Start Flex DNA Polymerase (NEB M0535). All forward primers were labeled at the 5′end with 6-carboxyfluorescein (FAM). Primer sequences and amplicon sizes for each gene are listed in Table 3. Purified recombinant *w*BmxR1 and *w*BmxR2 proteins (0.2 µg) were incubated with each DNA probe (0.1 pmol) for 10 min at room temperature in a 10 µl reaction mixture containing 10 mM Tris-HCl (pH 7.9), 10 mM MgCl_2_, 50 mM NaCl, 1 mM DTT, 0.1% NP-40 and 0.1 mg/mL calf thymus DNA (Sigma). Samples were then loaded onto 6% DNA retardation gels (Invitrogen) in 0.5×TBE buffer and run at 100 V for 2.5 h at 4°C. FAM-labeled DNA was detected in gels using Typhoon 9400 variable mode imager (GE Healthcare).

### Defining the minimal binding sequence of *ribA* promoter to *w*BmxR1

Gel shift assays were used to define the minimal binding sequence of the *ribA* promoter region to *w*BmxR1. Various sets of primers (Table 4) were designed to amplify specific regions of the promoter. Either the forward or reverse primer was labeled at the 5′ end with FAM. To generate double-stranded DNA from synthesized single-stranded oiligonucleotides, three pairs of complementary oligonucleotide primers were also synthesized and labeled with FAM. Complementary pairs of single strand oligonucleotides (60 nmol of each) were first heated in a heat block at 95°C for 5 min, annealed at room temperature for 1 hour to produce a double stranded DNA probe *in vitro* in a 50 µl reaction containing 10 mM Tris-HCl (pH7.5), 1 mM EDTA, and 50 mM NaCl. These double-stranded DNA probes were stored at −20°C until use. 0.1 µl of each probe was used in gel shift assays. Binding specificity for each labeled probe was demonstrated using a 50-fold excess of the corresponding unlabeled probe.

**Table 4:** Primers used to identify minimal binding region in *ribA* promoter.

Primer	Direction	Sequence 5′ to 3′	Amplicon size (bp)
A1	forward	TGTCTGGAATAACCTGAGACCACG	318
	reverse	TCTACCAATTTCACCACGACCGCA	
A2	forward	TGTCTGGAATAACCTGAGACCACG	238
	reverse	TCTAGTATCAGATTGACACTTTAGATGA	
A3	forward	TCACAAACTGAATTATACAGGCAATTCT	295
	reverse	GCCTAATTTCGCTGATGGCCCTTT	
A4	forward	TCTAGTATCAGATTGACACTTTAGATGA	259
	reverse	GCCTAATTTCGCTGATGGCCCTTT	
A5	forward	CTAAAATGATTAAGTAATTTAAAATGATTATATAAAAACTAG	225
	reverse	GCCTAATTTCGCTGATGGCCCTTT	
A6	forward	TCGTGGTGAAATTGGTAGACACGCAG	171
	reverse	GCCTAATTTCGCTGATGGCCCTTT	

### Construction of *lacZ* reporter fusions and β-galactosidase assay

Forward primer gagaGAATTCTGTCTGGAATAACCTGAGACCACG (EcoRI site underlined, additional nucleotides added to facilitate restriction enzyme digestion in lower case) and reverse primer gagaGGATCCGCCTAATTTCGCTGATGGCCCTTT (BamHI site underlined) were used to amplify the promoter region of *ribA* from *B. malayi* genomic DNA. The DNA fragment was subsequently cloned into pNK1415 [33] to create a transcriptional fusion vector *ribAp-lacZ*-pNK1415 with *lacZ* fusion driven by the *ribA* promoter. The USER cloning method (NEB) was then utilized to clone the *ribAp-lacZ* fusion into low copy number plasmid pACYC184 (NEB). The pACYC184 backbone was amplified using *PfuTurbo* C_x_ hotstart DNA polymerase (Agilent Technologies) with the primers actttcccUCCAGCAATAGACATAAGCGGCTAT and atgtctccgUTCTTCTTGAGATCGTTTTGGTCTGC derived from pACYC184. The *ribAp-lacZ* fusion was amplified with primers acggagacaUCGCTCTGCCGGTGGTTACCA and agggaaagUAACCTATAAAAATAGGCGTATCACGAGG derived from the *ribAp-lacZ*-pNK1415 construct. 10 μl of the *ribAp-lacZ* insert and 1 μl of the pACYC184 backbone were then incubated with 1 μl of USER enzyme at room temperature for 15 min before being transformed into *E. coli* cloning strain C3019 (NEB) to generate the *ribAp-lacZ*-pACYC184 reporter plasmid.

Both *ribAp-lacZ*-pACYC184 and *wBmxR1-*pET21a or *wBmxR2*-pET21a were transformed into *E. coli* strain C2566 (NEB) for measuring β-galactosidase activity measurement. The pET21a vector alone was used as a negative control. After overnight culture, transformants were sub-cultured at 37°C for 2 hours followed by induction with 0.1 mM IPTG for 3 hours. β–galactosidase activity was determined using a Miller Assay [34].

### Expression of genes of the vitamin B2 (riboflavin) pathway in *w*Bm

The vitamin B2 biosynthesis pathway was reconstructed based on the KEGG database (http://www.genome.jp/kegg). Enzymes that form the pathway include: GTP cyclohydrolase II, pyrimidine deaminase/reductase, 3.4-DHBP synthase, riboflavin synthase α and β chain. The corresponding ORF names in *Wolbachia* were found based on blast search. To determine gene expression, forward and reverse primers were designed (listed in Table 1) and used in RT-PCR reactions. Female *B. malayi* adult RNA was used to generate cDNA and used as template.

### Culture and treatment of adult *B. malayi* female worms

Living *B. malayi* adult female worms were purchased from TRS labs. Worms were washed extensively with RPMI1640 medium prior to culture in RPMI1640 medium supplemented with 2 mM glutamine, 10% Fetal Calf Serum (Gibco) and 100 U/mL streptomycin, 100 mg/mL penicillin, 0.25 mg/mL amphotericin B (Sigma). Four worms were distributed into each well of a 6-well plate and incubated at 37°C, in 5% CO_2_. After overnight recovery, worms were separated into 4 different groups each containing 12 worms (4 worms/well). Worms incubated in culture media alone (control group) or in culture media containing 0.5 µM doxycycline, 0.5 µM doxycycline plus 10 µg/mL vitamin B2, or 0.5 µM doxycycline plus 2 µg/mL vitamin B2. Culture media were changed daily. Adult worm motility and microfilaria production were recorded daily as described [35]. Motility was scored and expressed as a % of the motility relative to the motility scored on day 0 of the experiment. Microfilariae production was determined at day 7 by counting the number of microfilaria present in 1 mL of spent culture medium. The data obtained from triplicate samples (4 worms in each sample) are expressed as a mean ± standard deviation. The experiment was repeated twice.

## Results

### Identification of two EcxR homologs in *w*Bm

**Figure 1:**
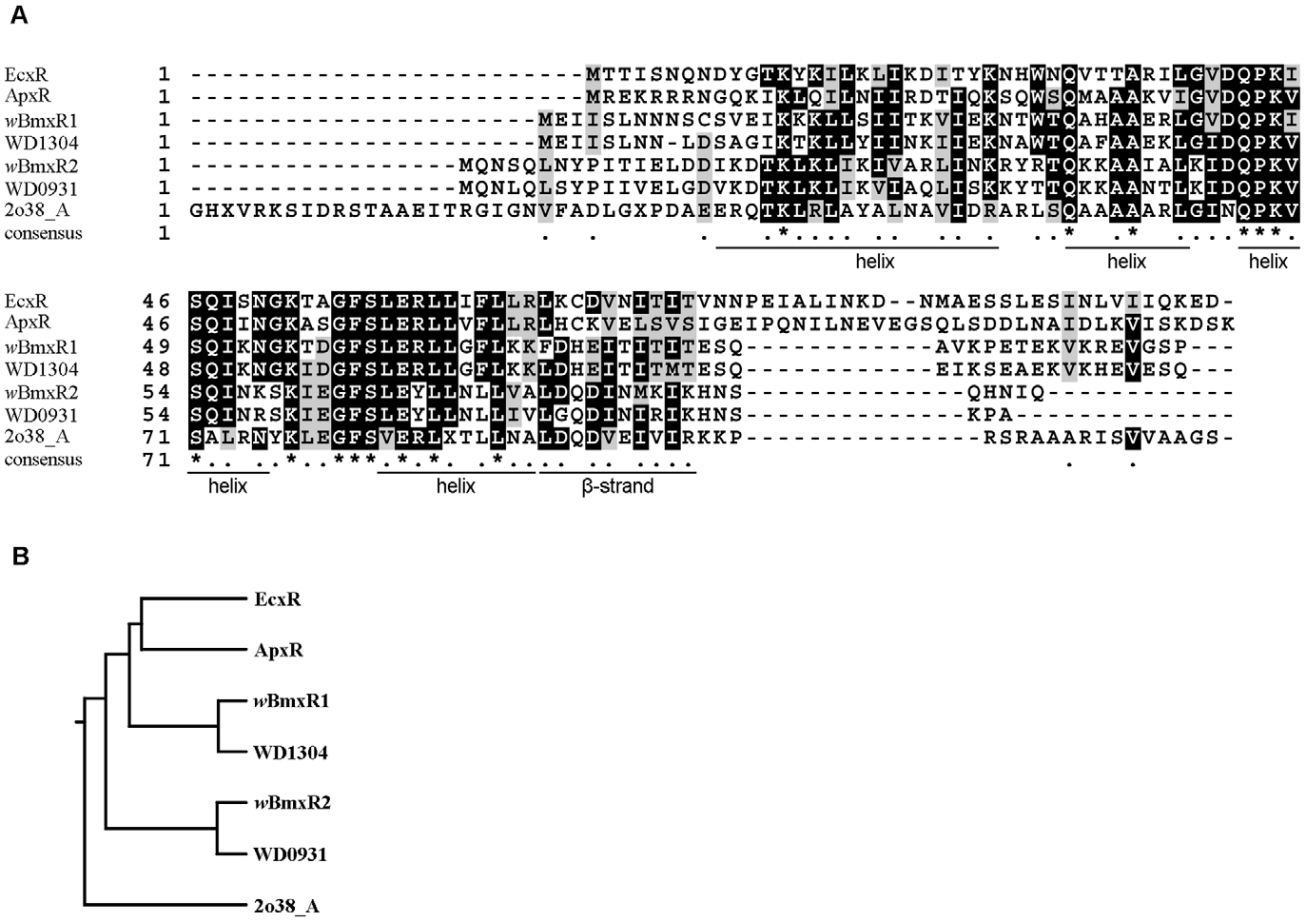
*Wolbachia* contains two potential homologs of the *Ehrlichia* type IV secretion system regulator EcxR. (A) Alignment of the deduced amino acid sequences of EcxR and homologs from *w*Bm (*w*BmxR1 and *w*BmxR2), *w*Mel (WD1304 and WD0931), *Anaplasma phagocytophilum* (ApxR), and *Rhodopseudomonas palustris* (2o83_A). The alignment was generated using TCOFFEE ClustalW (http://tcoffee.vital-it.ch/cgi-bin/Tcoffee/tcoffee_cgi/index.cgi) and displayed with BOXSHADE (www.ch.embnet.org/software/BOX_form.html). Identical (shaded black) or conserved (grey) amino acids present in at least two of the seven sequences are indicated. The positions of amino acids predicted to form a helix structure and β-strand are shown below the alignment and modeled using the 2o83_A structure. (B) Phylogenetic tree analysis of EcxR and various homologs. Branch length was generated using ClustalW (http://align.genome.jp/sit-bin/clustalw).

Using the EcxR sequence (YP_507593.1) to query NCBI databases, two potential homologs, *w*BmxR1 (wBm0386, YP_198216) and *w*BmxR2 (wBm0498, YP_198328.1), were identified in *Wolbachia* from *B. malayi*. *w*BmxR1 and *w*BmxR2 are 300 and 283 bp in length respectively, encoding 99 and 93 amino acid protein, each with a predicted molecular mass of 11 kDa. Two orthologs WD 1304 (NP_967012.1) and WD 0931 (NP_966668.1) were also found in *Wolbachia* of *Drosophila melanogaster* (*w*Mel). In addition, a structural ortholog, 2o38_A (gi 122921355), was identified with 99% confidence using the PHYRE program. 2o38_A is a putative XRE family transcriptional regulator present in the alphaproteobacterium *Rhodopseudomonas palustris*. Alignment of the deduced amino acid sequences of the various orthologs is shown in Fig. 1A. The previously characterized transcription factor ApxR (YP_505110.1) present in *Anaplasma phagocytophilum* [36], which is an ortholog of EcxR [29] was also included in the analysis. Structural analyses indicated that all orthologs share a conserved Helix–Turn–Helix domain that is present in a large family of alpha-helical proteins with a characteristic fold that functions as a sequence-specific DNA binding domain, such as in transcription regulators [37].

Phylogenetic analysis (Fig. 1B) indicates that *w*BmxR1 and WD1304 are closely related to each other, sharing 77% identity, while *w*BmxR2 and WD0931 share 76% identity to each other. *w*BmxR1 is more related to the previously characterized transcription factors, EcxR and ApxR, than *w*BmxR2. *w*BmxR1 and *w*BmxR2 share 45% identity to each other and also share 37% and 44% identity to 2o38_A.

### Analysis of *wBmxR1* and *wBmxR2* expression during the life cycle of *B. malayi*

**Figure 2:**
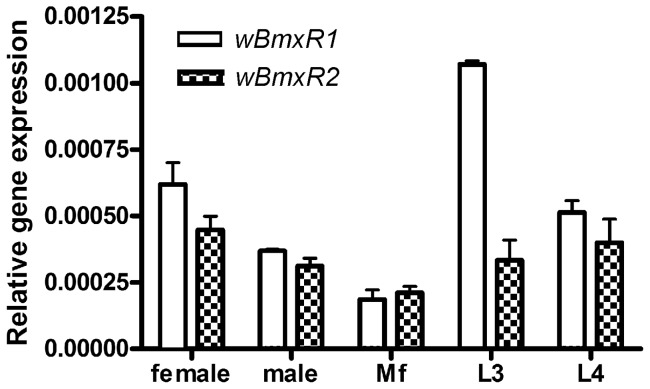
*Wolbachia wBmxR1* and *wBmxR2* are expressed in various developmental stages of *B. malayi*. RNA from adult female, male, microfilaria, L3 and L4 stages were analyzed by quantitative RT-PCR. The relative gene expression of *wBmxR1* and *wBmxR2* was represented relative to *Wolbachia* 16S rRNA. Data were obtained from triplicate samples and expressed as a mean ± standard deviation.

*Wolbachia* have been identified in all developmental stages of *B. malayi*, from studies on individual worms and isolates from regions endemic for lymphatic filariasis [38], [39], [40]. To determine the relative expression of *wBmxR1* and *wBmxR2* throughout the parasite life cycle, *wBmxR1* and *wBmxR2* mRNA expression was analyzed by quantitative real-time reverse transcription polymerase chain reaction (qRT-PCR). Relative levels of *wBmxR1* and *wBmxR2* expression (ratio of *wBmxR1* and *wBmxR2* to 16S rRNA) were calculated for each RNA sample (Fig. 2). *wBmxR1* and *wBmxR2* were transcribed in all stages examined (adult female and male worms, microfilariae, third- and fourth-stage larvae) with lowest levels in microfilariae. The expression level of *wBmxR1* is comparable to *wBmxR2*, except in third-stage larvae where *wBmxR1* showed 3-fold more expression than *wBmxR2*. No background was recorded in the assay using samples processed in the absence of reverse transcriptase, indicating the signal obtained was derived from RNA.

### Expression of VirB8 indicates that the Type IV secretion system in *w*Bm is active

**Figure 3:**
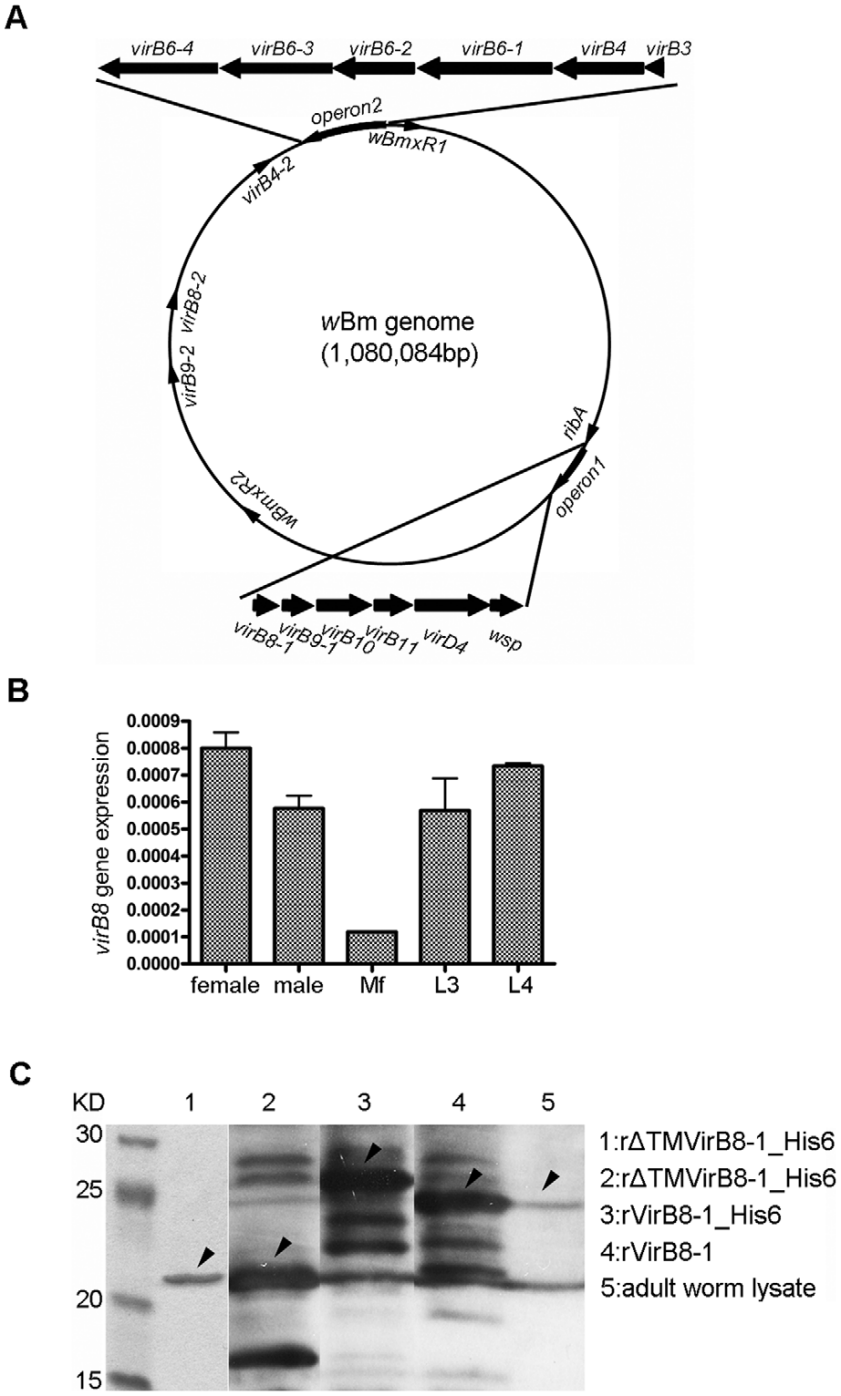
The Type IV secretion system of *w*Bm. (A) Schematic genome map of *w*Bm shown as a circle. The relative locations (shown as closed arrows) of operon 1 (*virB8-1, virB9-1, virB10, virB11, virD4, wsp*) and the upstream gene *ribA*, operon 2 (*virB3, virB4-1, virB6-1, virB6-2, virB6-3*, and *virB6-4*), *virB4-2*, *virB8-2*, *virB9-2*, *wBmxR1*, and *wBmxR2* are shown. (B) Expression of *Wolbachia virB8-1* in various developmental stages of *B. malayi.* RNA from adult female, male, microfilaria, L3 and L4 stages were analyzed by quantitative RT-PCR. The relative gene expression of *virB8-1* is represented relative to *Wolbachia* 16S rRNA. Data obtained from triplicate samples are expressed as a mean ± standard deviation. (C) Detection of VirB8-1 in protein lysates of adult female worms using anti-VirB8-1 antibody in Western blot analysis. Lane 1, SDS-PAGE gel showing purified recombinant VirB8-1 with an N-terminal His tag and deleted trans-membrane domain (rHis-ΔTM VirB8-1). Western blot analysis using rabbit serum generated against rHis-ΔTM VirB8-1 (lanes 2–5). Lane 2, lysate of *E. coli* expressing rHis-ΔTM VirB8-1; Lane 3, recombinant full length VirB8-1 with N-terminal His tag (rHis-VirB8-1); Lane 4, recombinant full length VirB8-1 minus tag (rVirB8-1); Lane 5, lysate of female *B. malayi* worms. Arrows indicate the band corresponding to the VirB8-1 protein.

Previous genome analysis has indicated the presence of a Type IV secretion system (T4SS) in *w*Bm with two operons and 3 individual genes encoding a total of 14 components. *Wolbachia* appear to lack certain components of the T4SS namely *virB2*, *virB5* and *virB7* [21], [22], [23], [24]. In *w*Bm, *virB8-1 (wBm0279)*, *virB9-1* (*wBm0280*), *virB10* (*wBm0281*), *virB11* (*wBm0282*) and *virD4* (*wBm0283*) are present in operon 1 (Fig. 3A). The *Wolbachia* surface protein encoded by *wsp* (*wBm0284*) is also contained in this operon. Operon 2 contains *virB3* (*wBm0798*), *virB4* (*wBm0797*), *virB6-1 (wBm0796*), *virB6-2* (*wBm0795*), *virB6-3* (*wBm0794*) and *virB6-4 (wBm0793*). Three duplicated genes: *virB4-2* (*wBm0750*), *virB8-2* (*wBm0641*) and *virB9-2* (*wBm0591*) are found scattered in the genome. The location of *wBmxR1 (wBm0386*) and *wBmxR2* (*wBm0498*) were also determined (Fig. 3A). A similar genomic organization is present in *E. chaffeensis*, except a *sodB* gene is the first transcribed gene in operon 2 rather than *virB3* [29]. In both *E. chaffeensis* and *w*Bm, *ribA* is located upstream of operon 1 (Fig. 3A).

The VirB8 protein is a critical component of the T4SS and is known to interact with other components of the system [41]. We demonstrated that the T4SS is expressed in *w*Bm since *virB8-1* is transcribed in adult female and male worms, microfilariae, third- and fourth-stage larvae, with lowest levels in microfilariae (Fig. 3B). Polyclonal rabbit sera raised against recombinant VirB8-1 (Fig. 3C, lane 1), reacted with lysates from *E. coli* expressing truncated his-tagged VirB8-1 protein (22 kDa, lane 2), full-length his-tagged protein (29 kDa, lane 3), and full-length VirB8-1 minus tag (27 kDa, lane 4). The additional bands observed may be due to protein degradation, but more likely due to antibody reactivity with *E. coli* proteins in the crude lysates as they were also found in un-induced *E. coli* lysates (data not shown). More importantly, the sera recognized a band of the expected size (27 kDa, lane 5) in lysates of adult female worms. The presence of *w*BmVirB8 indicates that the T4SS is likely active.

### Recombinant *w*BmxR1 and *w*BmxR2 bind to several promoter regions of *w*Bm T4SS

**Figure 4:**
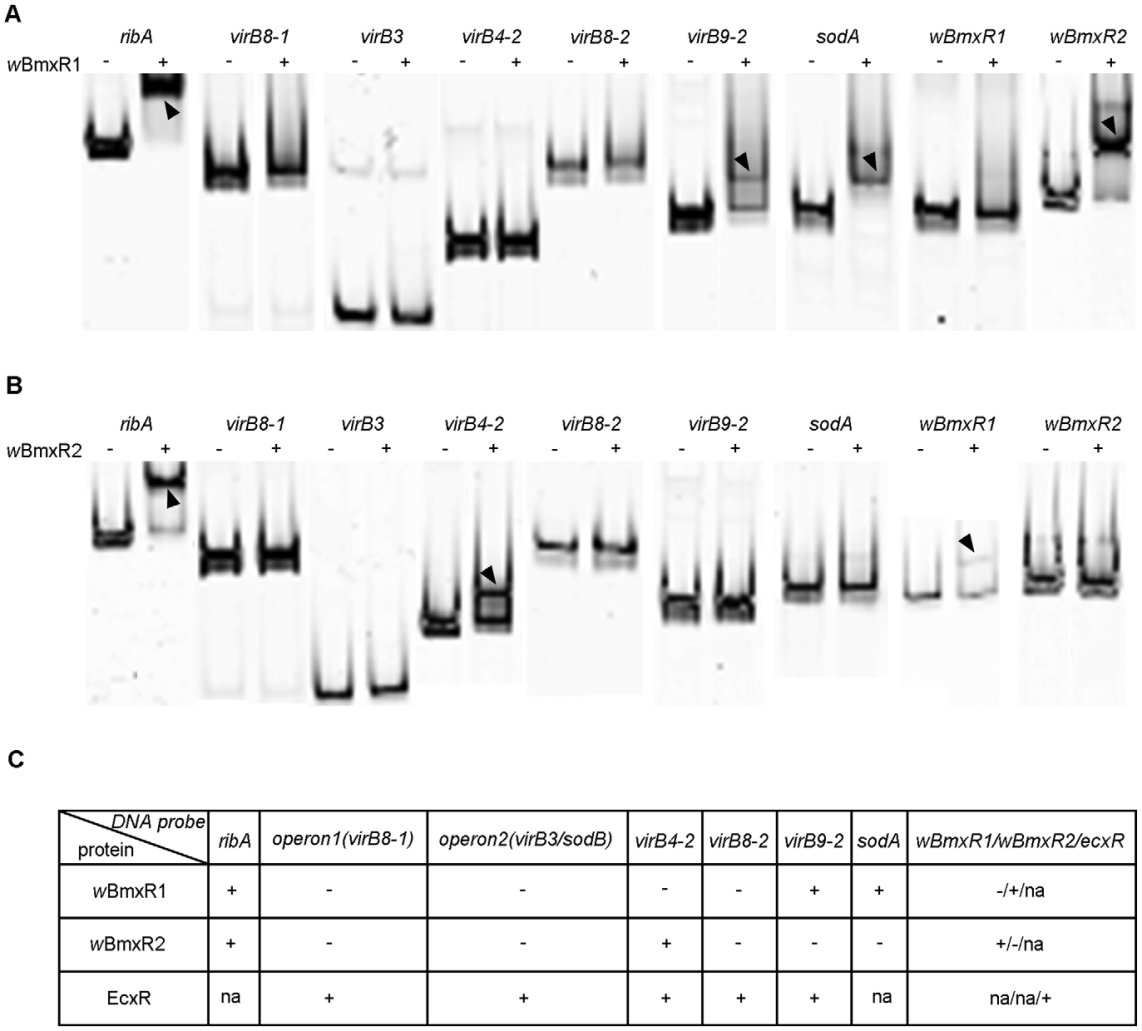
*w*BmxR1 and *w*BmxR2 bind the upstream promoter region of several genes. In electrophoretic mobility shift assays (EMSA), FAM-labeled DNA probes derived from the upstream region of *ribA*, *virB8-1*, *virB3*, *virB4-2*, *virB8-2*, *virB9-2*, *sodA*, *wBmxR1* and *wBmxR2*, and were incubated with (+) or without (–) protein and loaded onto a 6% DNA retardation gel. (A) EMSA using *w*BmxR1. (B) EMSA using *w*BmxR2. Arrowheads indicate shifted bands. Bands were visualized using a Typhoon scanner. (C) Summary of the EMSA of *w*BmxR1 and *w*BmxR2. The EMSA data from the previously characterized EcxR is included for comparison [29]. Positive gel shift is shown as ‘+’; no gel shift ‘−’; ‘na’, not applicable.

EcxR binds to five promoter regions of the *E. chaffeensis* T4SS, located in operon 1 (*virB8-1*), operon 2 (*sodB*), *virB4-2, virB8-2 and virB9-2* [29]. To determine whether *w*BmxR1 and *w*BmxR2 bind to the corresponding promoter regions of *w*Bm, gel shift assays were performed. In addition, the promoter regions upstream of *ribA* (*wBm0278*), *sodA* (*wBm0220*), *wBmxR1* and *wBmxR2* were included in the analysis (Fig. 4).

DNA probes derived from the sequence upstream of nine genes were generated by PCR using primers in Table 3 and labeled with FAM at the 5′ end. Electrophoretic mobility shifts assays (EMSA) showed *w*BmxR1 binds to regions upstream of the *virB9-2*, *ribA*, *sodA* and *wBmxR2* genes (Fig. 4A, C), whereas *w*BmxR2 binds to *virB4-2*, *ribA*, and the *wBmxR1* promoter regions (Fig. 4B, C). No binding was observed with either protein to the promoter region upstream of *virB3, virB8-1*, or *virB8-2* genes (Fig. 4A and B). Binding of both *w*BmxR1 and *w*BmxR2 to the same promoter region was only observed upstream of the *ribA* gene.

The results of the EMSA studies are summarized in Fig. 4C, and the published data on EcxR is included for comparison. Several differences were observed between *E. chaffeensis* [29] and *Wolbachia*. In *E. chaffeensis*, EcxR binds to the promoter of the predicted first gene in operon 1, namely *virB8-1*, whereas both *w*BmxR1 and *w*BmxR2 bind to the promoter region of the *ribA* gene that is located upstream of *virB8-1* in *w*Bm. EcxR also binds the promoter of the *sodB* gene (first gene in operon 2) that encodes superoxide dismutase, while in *Wolbachia*, *sodA* (not located in an operon), encodes superoxide dismutase, and its promoter region was found to bind *w*BmxR1. In addition, EcxR binds to its own promoter (*ecxR*). However, neither *w*BmxR1 nor *w*BmxR2 bound its own promoter. Instead, *w*BmxR1was found to bind to the promoter upstream of *wBmxR2* (Fig. 4A, C), while *w*BmxR2 binds to the promoter upstream of *wBmxR1* (Fig. 4B, C).

### *ribA* located upstream of operon 1 is co-transcribed with *virB8-1*

**Figure 5:**
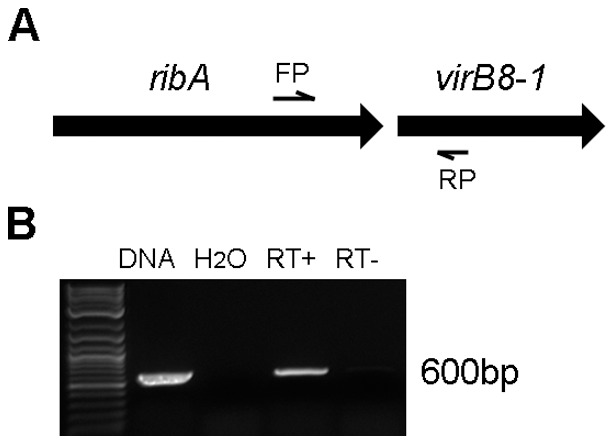
Detection of intergenic region between *ribA* and *virB8-1* by RT-PCR. cDNA from adult female *B. malayi* worms was used as the template in PCR reactions. The relative location of the primers (FP, RP) used to detect the intergenic region is shown. (B) Agarose gel showing PCR product resulting from amplification of intergenic region between *ribA* and *virB8-1*. Genomic DNA, water, and reverse transcriptase-minus (RT−) samples were included as controls.

Since *w*BmxR1 and *w*BmxR2 bind upstream of *ribA* but not *virB8-1*, we explored the possibility that *ribA* is co-transcribed with *virB8-1*. Using an operon prediction tool (http://www.microbesonline.org), *ribA* and *virB8* were predicted to be located in the same operon in *w*Bm but not in *E. chaffeensis.* RT-PCR experiments were then performed to determine if the *ribA* and *virB8-1* genes are co-transcribed as one operon in *w*Bm. A forward primer corresponding to *ribA* and reverse primer corresponding to *virB8-1* were used to specifically amplify the intergenenic region between *ribA* and *virB8-1* (Fig. 5A). A 600 bp product was observed using cDNA as a template (Fig. 5B, RT+). No DNA contamination was detected as no amplification was obtained using templates processed in the absence of reverse transcriptase (Fig. 5B, RT−). Therefore in *w*Bm, *ribA* located upstream of operon 1 is co-transcribed with *virB8-1*.

### The minimal region upstream of *ribA* that binds *w*BmxR1

**Figure 6:**
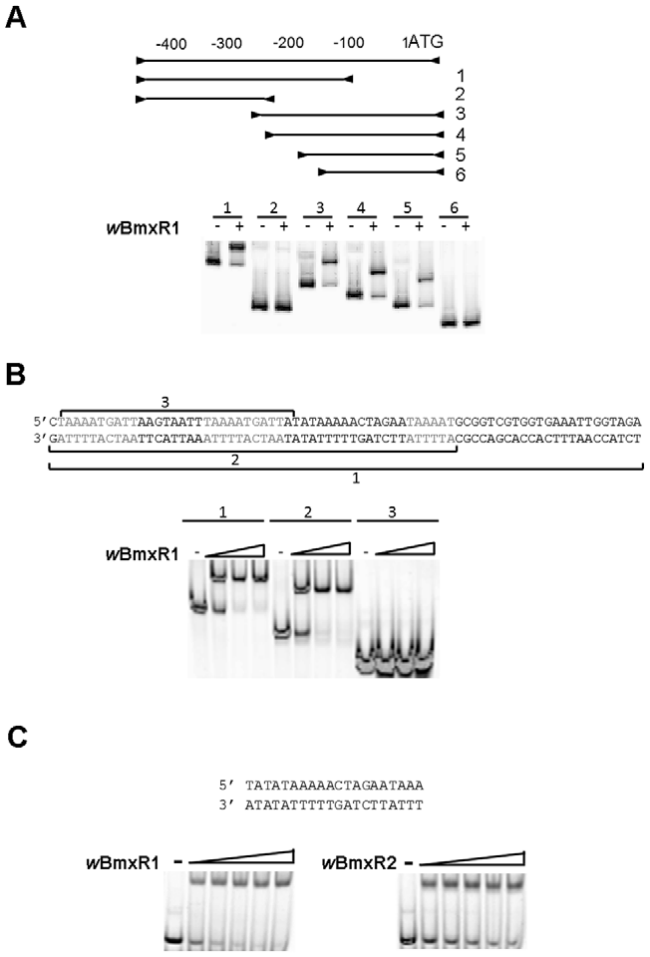
Identification of the minimal region upstream of *ribA* that binds *w*BmxR1. The minimal sequence of the *ribA* promoter region that binds *w*BmxR1 was identified an electrophoretic mobility shift assay (EMSA). (A) Six primer sets (A1 to A6 in Table 3) were used to generate six PCR products corresponding to various regions (from the ATG start site of *ribA* to 469 bp) upstream of the *ribA* promoter. Each PCR product was incubated with (+) or without (−) protein and loaded onto a 6% DNA retardation gel. (B) Three synthesized 5′FAM-labeled oligos (sequence shown) were annealed to dsDNA and used to shift *w*BmxR1. (C) *w*BmxR2 also shifts the minimum binding sequence for *w*BmxR1 in EMSA.

Experiments were then performed to determine the minimal DNA sequence upstream of *ribA* that is required for binding of *w*BmxR1. Six DNA fragments corresponding to various regions of the 469 bp promoter region of *ribA* (Fig. 6A) were generated using a series of specific primers (Table 4) and evaluated in EMSA. Four fragments resulted in positive gel shifts, indicating that binding occurred between –201 to –113 of the promoter (Fig. 6A). To refine the sequence further, three pairs of complementary oligonucleotide primers were then synthesized and annealed to produce various lengths of double-stranded DNA corresponding to the region between –201 to –113 (Fig. 6B). A 50 nt containing probe (probe 2, Fig. 6B) was sufficient to cause positive results in EMSA. Additional mapping of this region from both 5′ and 3′ ends of probe 2, identified a minimal 20 nt long binding sequence (TATATAAAAACTAGAATAAA), located 109 nt upstream of the ATG codon of *ribA*. *w*BmxR2 was also found to bind to the same sequence but with less affinity (Fig. 6C). The minimal binding sequence was then used to query the *w*Bm genome to identify additional promoter regions. Several sequences sharing various levels of homology were found, but none were located in predicted promoter regions.

### Recombinant *w*BmxR1 and *w*BmxR2 positively regulate a *ribAp*: *lacZ* reporter fusion

**Figure 7:**
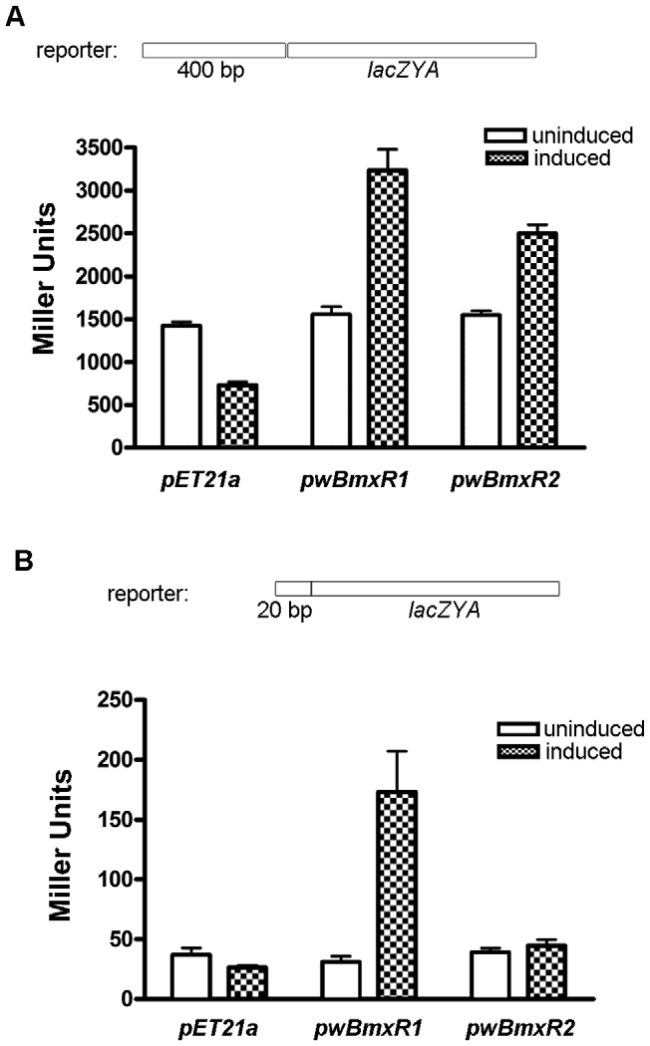
*w*BmxR1 and *w*BmxR2 positively regulate the transcription of *ribA lacZ* reporter. β–galactosidase assays were used to measure the transcriptional activities of *lacZ* reporter constructs. *E. coli* strain C2566 transformed with the reporter plasmid and protein expression vector *pET21a*, or *pwBmxR1*, or *pwBmxR2* were tested. β–galactosidase assays were performed on induced (IPTG) and un-induced samples. Miller units are shown as mean ± standard deviations from 3 replica experiments. The *ribA* promoter region containing 400 bp (A) or 20 bp minimal binding sequence (B) were fused to a promoter-less *lacZ* and cloned into low copy plasmid pACYC184 to generate the reporter plasmid.

In order to determine if *w*BmxR1 and *w*BmxR2 can activate the expression of *ribA*, a *lacZ* reporter fusion was constructed by inserting the promoter region (400 bp) of *ribA* upstream of the translation start of the promoter-less lacZ gene in pACYC184 (Fig. 7A). β-galactosidase assays were used to measure the transcriptional activity of the *lacZ* reporter. Following IPTG induction, a significant increase in β-galactosidase activity was detected in the presence of either *w*Bm protein, compared with uninduced samples, or samples prepared from empty vector alone. Western blotting experiments confirmed expression of *w*BmxR1 and *w*BmxR2 was only detected following induction with IPTG (data not shown). *lacZ* reporter fusions were also constructed to determine if *w*BmxR1 and *w*BmxR2 can activate expression of *virB9-2*/*sodA*, and *virB4-2*/*wBmxR1*, respectively. No activation or repression was observed (data not shown).

Additional experiments were performed to determine if *w*BmxR1 and *w*BmxR2 can activate the expression of *ribA* using the minimal binding sequence identified above (TATATAAAAACTAGAATAAA) fused to the promoter-less lacZ gene (Fig. 7B). Background levels of β-galactosidase activity were substantially lower, and induction of *w*BmxR1 with IPTG resulted in a significant increase in β-galactosidase activity. However in this case, *w*BmxR2 which binds this sequence with less affinity (Fig. 6C), did not activate the reporter.

### Riboflavin (Vitamin B2) synthesis pathway in *w*Bm

**Figure 8:**
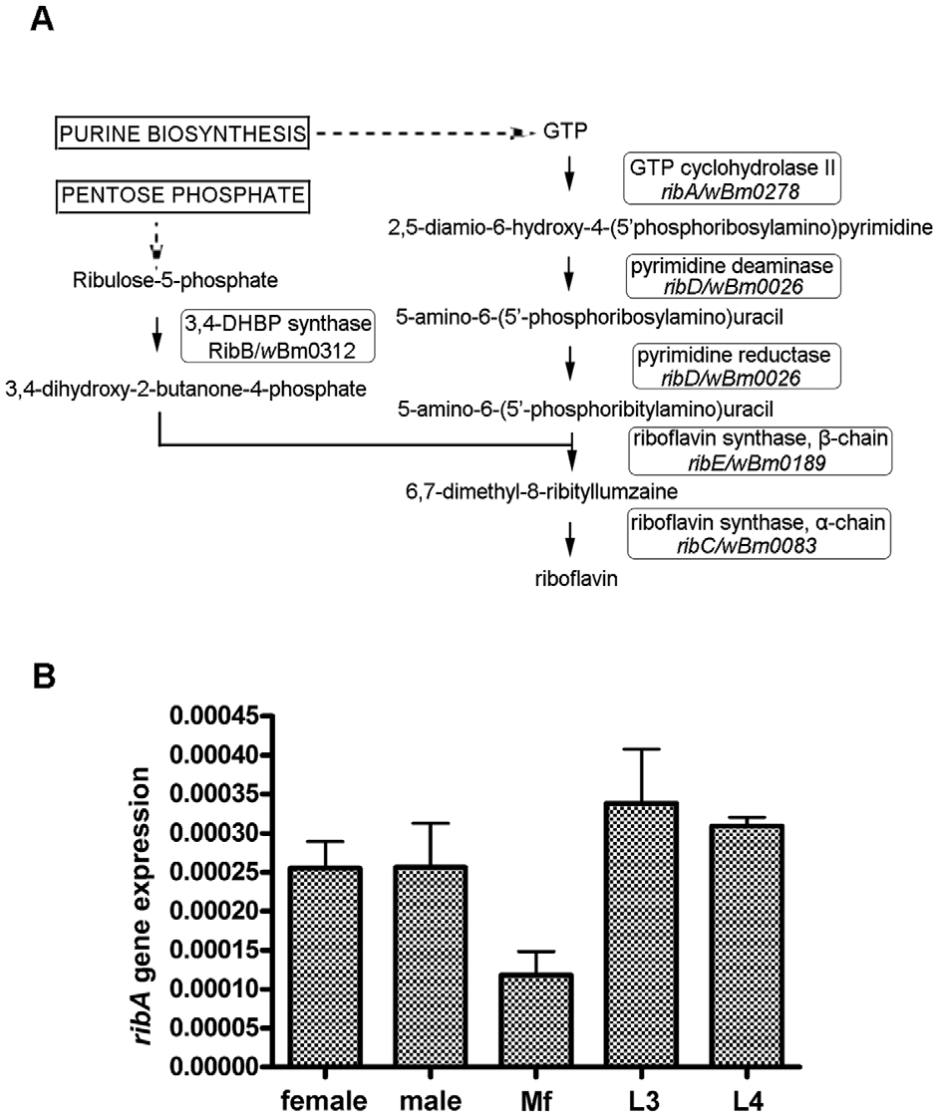
Vitamin B2 biosynthesis pathway in *w*Bm. (A) Schematic diagram of the riboflavin (vitamin B2) biosynthetic pathway. The various enzymes involved in the pathway and their corresponding genes in *E. coli* and *w*Bm are shown. (B) *Wolbachia ribA* gene expression in various developmental stages of *B. malayi*. RNA from adult female, male, microfilaria, L3 and L4 stages were analyzed. *ribA* expression is represented relative to *Wolbachia* 16S rRNA. Data obtained from triplicate samples are expressed as a mean ± standard deviation.

*RibA* encodes a bifunctional enzyme (3,4-dihydroxy-2-butanone-4-phosphate synthase and GTP cyclohydrolase II) which catalyzes two essential steps in riboflavin (vitamin B2) biosynthesis. In addition to *ribA*, we identified the remaining four genes in the pathway namely: *ribD* (*wBm0026*), *ribE* (*wBm0083*), *ribC* (*wBm0185*), *ribB* (*wBm0312*) indicating that the entire pathway is present in *w*Bm (Fig. 8A). Further analysis was performed on *ribA* to determine the relative expression of the gene throughout the parasite life cycle. qRT-PCR showed expression of *ribA* (ratio of *ribA* to 16S rRNA) in all stages examined (adult female and male worms, microfilariae, third- and fourth-stage larvae) with lowest levels in microfilariae (Fig. 8B). RT-PCR experiments using adult female *B. malayi* RNA showed *ribD*, *ribE*, and *ribB* genes are also expressed (data not shown).

### *Wolbachia* likely provide Vitamin B2 to *B. malayi* host

Genome analysis has indicated that *B. malayi* lack the entire riboflavin pathway [42] and it was predicted that a likely source of this essential nutrient is *w*Bm [43]. In order to determine if expression of the Vitamin B2 pathway described above (Fig. 8A) is consistent with an important nutritional role of *Wolbachia* for the nematode host, *B. malayi* worms were cleared of *Wolbachia* infection in culture using doxycycline and then supplemented with vitamin B2 to evaluate if any of the effects of drug treatment could be rescued. It has been shown that elimination of *Wolbachia* from *B. malayi* can block embryogenesis and cause parasite death [6], [44], [45].

**Figure 9:**
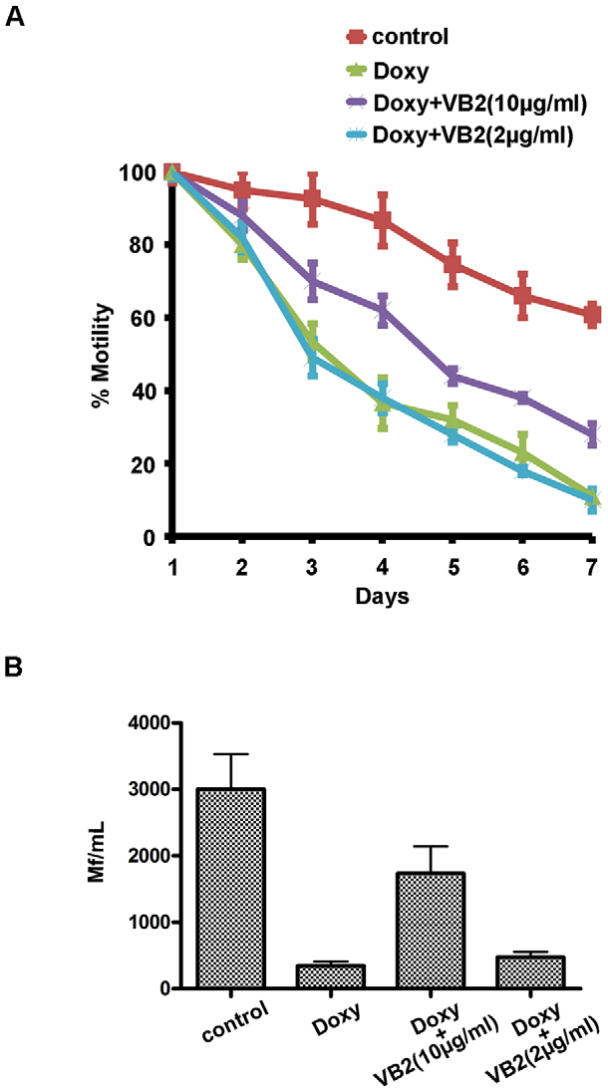
Vitamin B2 improved motility of *B. malayi* worms and microfilarial production following doxycycline treatment. Adult female *B. malayi* were cultured in the absence (control) or presence of 0.5 µM doxycycline, or antibiotic supplemented with either 2 µg/mL or 10 µg/mL vitamin B2. The motility of worms *in vitro* was examined daily for 7 days (A). Microfilariae production was determined at day 7 by counting the number of microfilaria present in 1 mL spent culture medium (B). Data obtained from triplicate samples (4 worms in each sample) are expressed as a mean ± standard deviation.

Adult female *B. malayi* were cultured in the absence or presence of 0.5 µM doxycycline, or antibiotic supplemented with either 2 µg/mL or 10 µg/mL vitamin B2 (Fig. 9A and 9B). Adult worm motility and microfilaria production were recorded daily as described [35]. Doxycycline treatment resulted in a rapid decrease in motility (Fig. 9A) and microfilaria production (Fig. 9B) compared to untreated control worms. When the culture media were supplemented with 10 µg/mL vitamin B2, adult worm motility and microfilaria production remained at approximately 50% of normal levels. The addition of 2 µg/mL vitamin B2 was not sufficient to rescue the motility (Fig. 9A) or fertility defects (Fig. 9B).

## Discussion

Secretion systems are known to play a number of critical roles in bacteria. As a result they are attractive as potential drug targets for the development of new antibiotics [46]. Many pathogenic intracellular bacteria and bacterial endosymbionts use a type IV secretion system (T4SS) to transport effector molecules (DNA, proteins or DNA-protein complexes) across the membrane into the cytoplasm or nucleus of eukaryotic cells. *Agrobacterium tumefaciens*, *Bartonella spp*., *Bordetella pertussis*, *Brucella* spp., *Helicobacter pylori*, and *Legionella pneumophila* use a T4SS to deliver virulence factors [17]. Amongst obligate intracellular bacteria, the T4SS has been characterized in Rickettsia-like bacteria such as *Anaplasma phagocytophila* and *Ehrlichia chaffeensis* [47], [48], [49]. It is present and highly conserved in insect [21], [22], [23], [24] and nematode *Wolbachia* [21], [50]. In *w*Bm, two operons and 3 individual genes (*virB4-2*, *virB8-2* and *virB9-2*) encode a total of 14 components. *VirB8-1*, *virB9-1*, *virB10*, *virB11* and *virD4* reside in operon 1, while *virB3*, *virB4*, *virB6-1*, *virB6-2*, *virB6-3* and *virB6-4* are found in operon 2. VirB8 is an essential assembly factor for all T4SS and has been considered a suitable target for drugs that inhibit its protein-protein interactions [41]. Recently, specific small molecule inhibitors of *Brucella abortus* VirB8 were identified in a high-throughput screen and one compound strongly inhibited bacterial proliferation *in vivo* [51]. Screening efforts to isolate T4SS inhibitors have also led to the discovery of compounds that inhibit *Helicobacter pylori* VirB11 ATPase activity [52] and the T4SS mediated transfer of broad-host-range plasmids [53]. We demonstrate here that the T4SS is likely active in *w*Bm since *virB8-1* is transcribed in adult female and male worms, microfilariae, third- and fourth-stage larvae. In addition, antibody generated against highly purified recombinant VirB8-1 recognizes a band of the expected size (27 kDa) in lysates of adult female worms. All other components of the *w*Bm T4SS except VirB9-2 have also been detected in proteomic analyses of different stages of *B. malayi* parasites [54], further supporting the likelihood that the T4SS is active. Therefore, the discovery of inhibitors of T4SS system of *w*Bm may lead to the development of novel compounds to treat filarial diseases.

Transcription factors that regulate the secretion system components are also attractive as potential drug targets for the development of new antibiotics [46]. In *Ehrlichia chaffeensis*, a regulator (EcxR) of the T4SS has been identified [29]. We identified two orthologs (*w*BmxR1 and *w*BmxR2) in *w*Bm and in the *Wolbachia* of *Drosophila melanogaster* (*w*Mel, WD1304 and WD0931). Our analysis of other *Wolbachia* genomes (*w*Ri, *Drosophila simulans*; *w*Pip, *Culex pipiens*; *w*Oo, *Onchocerca ochengi*; wLs, *Litomosoides sigmodontis* and *w*Di, *Dirofilaria immitis*) revealed the presence of orthologs of both *w*BmxR1 and *w*BmxR2 (data not shown). While the majority of *w*Bm genes are expressed in a stage-specific manner [54], *wBmxR1* and *wBmxR2* were found to be expressed in both male and female worms as well as in all larval stages examined, which is consistent with important roles for T4SS and vitamin B2 in the biology of *Wolbachia*. In proteomic analyses of adult worms and microfilariae of *B. malayi* (not enriched for *Wolbachia*), *w*BmxR1 has also been detected [54].

Interestingly, *w*BmxR1 and *w*BmxR2 were found to regulate certain genes of the T4SS and *ribA* located upstream of *virB8-1*. *RibA* encodes a bifunctional enzyme that catalyzes two essential steps in riboflavin (vitamin B2) biosynthesis. Vitamin B2 is an obligatory supplement of human and animal diets, as it serves as the precursor of flavin coenzymes, flavin mononucleotide, and flavin adenine dinucleotide, which are involved in oxidative metabolism and other processes [55]. *ribA* is present in the *virB8-D4* operon in all *Wolbachia* strains, and also in several *Ehrlichia* and *Anaplasma* genomes suggesting that the *ribA-virD4* locus may have been present in their common ancestor and important for the integrity and transcriptional activity of the *virB8–D4* operon in *Anaplasma*, *Ehrlichia* and *Wolbachia* [24]. Other genes of interest in the *virB8–D4* locus include *wspB* which encodes a *Wolbachia* surface antigen [24]. *wspB* has also been shown to be co-transcribed as part of the *virB8–D4* operon in 3 *Wolbachia* strains belonging to A- and B-supergroups [23], [56]. These studies and our data demonstrate that regulation of the T4SS is variable and complex in different organisms. In *w*Bm, the transcription factors *w*BmxR1 and *w*BmxR2 were found to regulate certain genes of the T4SS and the vitamin B2 biosynthesis pathway. Since *w*BmxR1 and *w*BmxR2 did not bind to the promoter regions of *virB8-2* or the *virB* genes located in operon 2, it is likely that there are other transcription factors involved in regulation of the T4SS in *w*Bm.

Analysis of the full genome sequence of *B. malayi* revealed that it lacks the biosynthetic pathway for vitamin B2 [42]. In the present study, we show that the pathway is complete and expressed in *w*Bm. Furthermore, depletion of *w*Bm led to a decrease in adult worm motility and microfilarial production, and the addition of vitamin B2 (10 µg/ml) to the culture partially rescued these effects. Genomic DNA was extracted from treated and untreated parasites and PCR was performed to measure the *Wolbachia* load. We did not observe a measurable difference between untreated, drug-treated, and vitamin B2 supplemented samples in the time frame of the experiment (data not shown). A similar rescue was reported previously in the *Wolbachia*/bedbug system, where elimination of *Wolbachia* in *Cimex lecturalis* using antibiotics resulted in retarded growth and sterility of the host insect. These deficiencies were rescued by supplementation of B vitamins including vitamin B2 (20 µg/ml) [57]. While the conditions used in these experiments may not exactly mimic the natural environment, these data indicate that *Wolbachia* strains may supply the essential vitamin to their hosts. Consistent with this is our finding of an intact vitamin B2 biosynthesis pathway in various insect *Wolbachia* namely: *w*Mel, *w*Ri and *w*Pip (data not shown). Similar analyses of the incomplete genome sequences available from the filarial *Wolbachia w*Ls and *w*Di, revealed that the pathway is likely functional with the exception of RibA in *w*Ls and RibC in *w*Di, other enzymes are present. Metabolic provisioning of hosts by endosymbionts is commonly observed in obligate associations [58], including the supply of vitamin B2 from *Buchnera aphidcola* to their insect hosts [59], [60], [61]. Regarding filarial parasites, *w*Bm has been shown to provide the essential cofactor heme to *B. malayi* [62].

While it is likely that several *Wolbachia* strains can supply vitamin B2 to their hosts, exceptions clearly exist since the recent sequencing of the genome of *Wolbachia* of *Onchocerca ochengi* (*w*Oo) has revealed the loss of many features including genes involved in cofactor metabolism, and genes of the riboflavin pathway are either lost or psueodgenized, with the exception of *ribA* [50]. It will be interesting to understand how *O. ochengi* obtains Vitamin B2. The same question can be applied to *Wolbachia*-free filarial parasites such as *Loa loa* and *Acanthocheilonema viteae* which are unable to synthesize vitamin B2 *de novo*. These parasites are presumably dependent on their host for vitamin B2 and other nutrients that may be supplied by the endosymbiont.

The use of antibiotics targeting the *Wolbachia* endosymbionts of filarial parasites has been validated as an approach for controlling filarial infection in animals and humans. Understanding the molecular and/or biochemical basis of the filaria–*Wolbachia* relationship and identification of important pathways and proteins involved are required for development of drugs that disrupt the symbiosis. In the case of *w*Oo, rather than nutrient or cofactor provisioning, transcriptome analyses has indicated potential roles of the endosymbiont in energy production and modulation of the mammalian immune response [50]. In our study, we have identified two transcription factors, *w*BmxR1 and *w*BmxR2, that co-regulate the T4SS and vitamin B2 pathway in *w*Bm. Given the likely important roles of these regulators in the symbiotic relationship, they warrant further investigation as potential drug targets for the development of new antibiotics for filarial infection.

## References

[1] Hoerauf A, Volkmann L, Hamelmann C, Adjei O, Autenrieth IB, et al (2000) Endosymbiotic bacteria in worms as targets for a novel chemotherapy in filariasis. Lancet 355: 1242–1243. 10770311 10.1016/S0140-6736(00)02095-X

[2] Hoerauf A, Mand S, Adjei O, Fleischer B, Buttner DW (2001) Depletion of wolbachia endobacteria in Onchocerca volvulus by doxycycline and microfilaridermia after ivermectin treatment. Lancet 357: 1415–1416. 11356444 10.1016/S0140-6736(00)04581-5

[3] Taylor MJ, Makunde WH, McGarry HF, Turner JD, Mand S, et al (2005) Macrofilaricidal activity after doxycycline treatment of Wuchereria bancrofti: a double-blind, randomised placebo-controlled trial. Lancet 365: 2116–2121. 15964448 10.1016/S0140-6736(05)66591-9

[4] Debrah AY, Mand S, Marfo-Debrekyei Y, Batsa L, Albers A, et al (2011) Macrofilaricidal Activity in Wuchereria bancrofti after 2 Weeks Treatment with a Combination of Rifampicin plus Doxycycline. J Parasitol Res 2011: 201617. 21687646 10.1155/2011/201617 PMC3112504

[5] Debrah AY, Mand S, Specht S, Marfo-Debrekyei Y, Batsa L, et al (2006) Doxycycline reduces plasma VEGF-C/sVEGFR-3 and improves pathology in lymphatic filariasis. PLoS Pathog 2: e92. 17044733 10.1371/journal.ppat.0020092 PMC1564427

[6] Debrah AY, Mand S, Marfo-Debrekyei Y, Batsa L, Pfarr K, et al (2007) Macrofilaricidal effect of 4 weeks of treatment with doxycycline on Wuchereria bancrofti. Trop Med Int Health 12: 1433–1441. 18076549 10.1111/j.1365-3156.2007.01949.x

[7] Hoerauf A, Marfo-Debrekyei Y, Buttner M, Debrah AY, Konadu P, et al (2008) Effects of 6-week azithromycin treatment on the Wolbachia endobacteria of Onchocerca volvulus. Parasitol Res 103: 279–286. 18421478 10.1007/s00436-008-0964-x

[8] Mand S, Pfarr K, Sahoo PK, Satapathy AK, Specht S, et al (2009) Macrofilaricidal activity and amelioration of lymphatic pathology in bancroftian filariasis after 3 weeks of doxycycline followed by single-dose diethylcarbamazine. Am J Trop Med Hyg 81: 702–711. 19815891 10.4269/ajtmh.2009.09-0155

[9] Jeyaprakash A, Hoy MA (2000) Long PCR improves Wolbachia DNA amplification: wsp sequences found in 76% of sixty-three arthropod species. Insect Mol Biol 9: 393–405. 10971717 10.1046/j.1365-2583.2000.00203.x

[10] Werren JH, Windsor DM (2000) Wolbachia infection frequencies in insects: evidence of a global equilibrium? Proc Biol Sci 267: 1277–1285. 10972121 10.1098/rspb.2000.1139 PMC1690679

[11] Bennett GM, Pantoja NA, O'Grady PM (2012) Diversity and phylogenetic relationships of Wolbachia in Drosophila and other native Hawaiian insects. Fly (Austin) 6. 10.4161/fly.21161 PMC3519662 22878693

[12] Zug R, Hammerstein P (2012) Still a host of hosts for Wolbachia: analysis of recent data suggests that 40% of terrestrial arthropod species are infected. PLoS One 7: e38544. 22685581 10.1371/journal.pone.0038544 PMC3369835

[13] Sunish IP, Rajendran R, Paramasivan R, Dhananjeyan KJ, Tyagi BK (2011) Wolbachia endobacteria in a natural population of Culex quinquefasciatus from filariasis endemic villages of south India and its phylogenetic implication. Trop Biomed 28: 569–576. 22433886

[14] McGraw EA, O'Neill SL (2004) Wolbachia pipientis: intracellular infection and pathogenesis in Drosophila. Curr Opin Microbiol 7: 67–70. 15036143 10.1016/j.mib.2003.12.003

[15] Narita S, Kageyama D, Nomura M, Fukatsu T (2007) Unexpected mechanism of symbiont-induced reversal of insect sex: feminizing Wolbachia continuously acts on the butterfly Eurema hecabe during larval development. Appl Environ Microbiol 73: 4332–4341. 17496135 10.1128/AEM.00145-07 PMC1932763

[16] Werren JH (1997) Biology of Wolbachia. Annu Rev Entomol 42: 587–609. 15012323 10.1146/annurev.ento.42.1.587

[17] Alvarez-Martinez CE, Christie PJ (2009) Biological diversity of prokaryotic type IV secretion systems. Microbiol Mol Biol Rev 73: 775–808. 19946141 10.1128/MMBR.00023-09 PMC2786583

[18] Llosa M, Roy C, Dehio C (2009) Bacterial type IV secretion systems in human disease. Mol Microbiol 73: 141–151. 19508287 10.1111/j.1365-2958.2009.06751.x PMC2784931

[19] Zechner EL, Lang S, Schildbach JF (2012) Assembly and mechanisms of bacterial type IV secretion machines. Philos Trans R Soc Lond B Biol Sci 367: 1073–1087. 22411979 10.1098/rstb.2011.0207 PMC3297438

[20] Christie PJ, Atmakuri K, Krishnamoorthy V, Jakubowski S, Cascales E (2005) Biogenesis, architecture, and function of bacterial type IV secretion systems. Annu Rev Microbiol 59: 451–485. 16153176 10.1146/annurev.micro.58.030603.123630 PMC3872966

[21] Pichon S, Bouchon D, Cordaux R, Chen L, Garrett RA, et al (2009) Conservation of the Type IV secretion system throughout Wolbachia evolution. Biochem Biophys Res Commun 385: 557–562. 19486895 10.1016/j.bbrc.2009.05.118

[22] Masui S, Sasaki T, Ishikawa H (2000) Genes for the type IV secretion system in an intracellular symbiont, Wolbachia, a causative agent of various sexual alterations in arthropods. J Bacteriol 182: 6529–6531. 11053403 10.1128/jb.182.22.6529-6531.2000 PMC94805

[23] Rances E, Voronin D, Tran-Van V, Mavingui P (2008) Genetic and functional characterization of the type IV secretion system in Wolbachia. J Bacteriol 190: 5020–5030. 18502862 10.1128/JB.00377-08 PMC2447017

[24] Felix C, Pichon S, Braquart-Varnier C, Braig H, Chen L, et al (2008) Characterization and transcriptional analysis of two gene clusters for type IV secretion machinery in Wolbachia of Armadillidium vulgare. Res Microbiol 159: 481–485. 18582562 10.1016/j.resmic.2008.05.007

[25] Martinez-Nunez C, Altamirano-Silva P, Alvarado-Guillen F, Moreno E, Guzman-Verri C, et al (2010) The two-component system BvrR/BvrS regulates the expression of the type IV secretion system VirB in Brucella abortus. J Bacteriol 192: 5603–5608. 20833814 10.1128/JB.00567-10 PMC2953682

[26] de Jong MF, Sun YH, den Hartigh AB, van Dijl JM, Tsolis RM (2008) Identification of VceA and VceC, two members of the VjbR regulon that are translocated into macrophages by the Brucella type IV secretion system. Mol Microbiol 70: 1378–1396. 19019140 10.1111/j.1365-2958.2008.06487.x PMC2993879

[27] Altman E, Segal G (2008) The response regulator CpxR directly regulates expression of several Legionella pneumophila icm/dot components as well as new translocated substrates. J Bacteriol 190: 1985–1996. 18192394 10.1128/JB.01493-07 PMC2258895

[28] Zusman T, Aloni G, Halperin E, Kotzer H, Degtyar E, et al (2007) The response regulator PmrA is a major regulator of the icm/dot type IV secretion system in Legionella pneumophila and Coxiella burnetii. Mol Microbiol 63: 1508–1523. 17302824 10.1111/j.1365-2958.2007.05604.x

[29] Cheng Z, Wang X, Rikihisa Y (2008) Regulation of type IV secretion apparatus genes during Ehrlichia chaffeensis intracellular development by a previously unidentified protein. J Bacteriol 190: 2096–2105. 18192398 10.1128/JB.01813-07 PMC2258868

[30] Kelley LA, Sternberg MJ (2009) Protein structure prediction on the Web: a case study using the Phyre server. Nat Protoc 4: 363–371. 19247286 10.1038/nprot.2009.2

[31] Mathews SA, Volp KM, Timms P (1999) Development of a quantitative gene expression assay for Chlamydia trachomatis identified temporal expression of sigma factors. FEBS Lett 458: 354–358. 10570939 10.1016/s0014-5793(99)01182-5

[32] Li Z, Garner AL, Gloeckner C, Janda KD, Carlow CK (2011) Targeting the Wolbachia cell division protein FtsZ as a new approach for antifilarial therapy. PLoS Negl Trop Dis 5: e1411. 22140592 10.1371/journal.pntd.0001411 PMC3226453

[33] Simons RW, Houman F, Kleckner N (1987) Improved single and multicopy lac-based cloning vectors for protein and operon fusions. Gene 53: 85–96. 3596251 10.1016/0378-1119(87)90095-3

[34] Miller MA, editor (1972) Experiments in Moleculary Genetics: Cold Spring Harbor Laboratory. 352–355 p.

[35] Rao RU, Moussa H, Weil GJ (2002) Brugia malayi: effects of antibacterial agents on larval viability and development in vitro. Exp Parasitol 101: 77–81. 12243742 10.1016/s0014-4894(02)00019-x

[36] Wang X, Kikuchi T, Rikihisa Y (2007) Proteomic identification of a novel Anaplasma phagocytophilum DNA binding protein that regulates a putative transcription factor. J Bacteriol 189: 4880–4886. 17483233 10.1128/JB.00318-07 PMC1913470

[37] Aravind L, Anantharaman V, Balaji S, Babu MM, Iyer LM (2005) The many faces of the helix-turn-helix domain: transcription regulation and beyond. FEMS Microbiol Rev 29: 231–262. 15808743 10.1016/j.femsre.2004.12.008

[38] Kozek WJ (1977) Transovarially-transmitted intracellular microorganisms in adult and larval stages of Brugia malayi. J Parasitol 63: 992–1000. 592054

[39] Taylor MJ, Bilo K, Cross HF, Archer JP, Underwood AP (1999) 16S rDNA phylogeny and ultrastructural characterization of Wolbachia intracellular bacteria of the filarial nematodes Brugia malayi, B. pahangi, and Wuchereria bancrofti. Exp Parasitol 91: 356–361. 10092480 10.1006/expr.1998.4383

[40] McGarry HF, Egerton GL, Taylor MJ (2004) Population dynamics of Wolbachia bacterial endosymbionts in Brugia malayi. Mol Biochem Parasitol 135: 57–67. 15287587 10.1016/j.molbiopara.2004.01.006

[41] Baron C (2006) VirB8: a conserved type IV secretion system assembly factor and drug target. Biochem Cell Biol 84: 890–899. 17215876 10.1139/o06-148

[42] Ghedin E, Wang S, Spiro D, Caler E, Zhao Q, et al (2007) Draft genome of the filarial nematode parasite Brugia malayi. Science 317: 1756–1760. 17885136 10.1126/science.1145406 PMC2613796

[43] Foster J, Ganatra M, Kamal I, Ware J, Makarova K, et al (2005) The Wolbachia genome of Brugia malayi: endosymbiont evolution within a human pathogenic nematode. PLoS Biol 3: e121. 15780005 10.1371/journal.pbio.0030121 PMC1069646

[44] Debrah AY, Mand S, Marfo-Debrekyei Y, Larbi J, Adjei O, et al (2006) Assessment of microfilarial loads in the skin of onchocerciasis patients after treatment with different regimens of doxycycline plus ivermectin. Filaria J 5: 1. 16457735 10.1186/1475-2883-5-1 PMC1388215

[45] Hoerauf A, Specht S, Marfo-Debrekyei Y, Buttner M, Debrah AY, et al (2009) Efficacy of 5-week doxycycline treatment on adult Onchocerca volvulus. Parasitol Res 104: 437–447. 18850111 10.1007/s00436-008-1217-8

[46] Baron C, Coombes B (2007) Targeting bacterial secretion systems: benefits of disarmament in the microcosm. Infect Disord Drug Targets 7: 19–27. 17346208 10.2174/187152607780090685

[47] Ohashi N, Zhi N, Lin Q, Rikihisa Y (2002) Characterization and transcriptional analysis of gene clusters for a type IV secretion machinery in human granulocytic and monocytic ehrlichiosis agents. Infect Immun 70: 2128–2138. 11895979 10.1128/IAI.70.4.2128-2138.2002 PMC127848

[48] Rikihisa Y, Lin M, Niu H (2010) Type IV secretion in the obligatory intracellular bacterium Anaplasma phagocytophilum. Cell Microbiol 12: 1213–1221. 20670295 10.1111/j.1462-5822.2010.01500.x PMC3598623

[49] Rikihisa Y, Lin M, Niu H, Cheng Z (2009) Type IV secretion system of Anaplasma phagocytophilum and Ehrlichia chaffeensis. Ann N Y Acad Sci 1166: 106–111. 19538269 10.1111/j.1749-6632.2009.04527.x

[50] Darby AC, Armstrong SD, Bah GS, Kaur G, Hughes MA, et al.. (2012) Analysis of gene expression from the Wolbachia genome of a filarial nematode supports both metabolic and defensive roles within the symbiosis. Genome Res. 10.1101/gr.138420.112 PMC3514676 22919073

[51] Paschos A, den Hartigh A, Smith MA, Atluri VL, Sivanesan D, et al (2011) An in vivo high-throughput screening approach targeting the type IV secretion system component VirB8 identified inhibitors of Brucella abortus 2308 proliferation. Infect Immun 79: 1033–1043. 21173315 10.1128/IAI.00993-10 PMC3067494

[52] Fernandez-Lopez R, Machon C, Longshaw CM, Martin S, Molin S, et al (2005) Unsaturated fatty acids are inhibitors of bacterial conjugation. Microbiology 151: 3517–3526. 16272375 10.1099/mic.0.28216-0

[53] Hilleringmann M, Pansegrau W, Doyle M, Kaufman S, MacKichan ML, et al (2006) Inhibitors of Helicobacter pylori ATPase Cagalpha block CagA transport and cag virulence. Microbiology 152: 2919–2930. 17005973 10.1099/mic.0.28984-0

[54] Bennuru S, Meng Z, Ribeiro JM, Semnani RT, Ghedin E, et al (2011) Stage-specific proteomic expression patterns of the human filarial parasite Brugia malayi and its endosymbiont Wolbachia. Proc Natl Acad Sci U S A 108: 9649–9654. 21606368 10.1073/pnas.1011481108 PMC3111283

[55] Abbas CA, Sibirny AA (2011) Genetic control of biosynthesis and transport of riboflavin and flavin nucleotides and construction of robust biotechnological producers. Microbiol Mol Biol Rev 75: 321–360. 21646432 10.1128/MMBR.00030-10 PMC3122625

[56] Wu M, Sun LV, Vamathevan J, Riegler M, Deboy R, et al (2004) Phylogenomics of the reproductive parasite Wolbachia pipientis wMel: a streamlined genome overrun by mobile genetic elements. PLoS Biol 2: E69. 15024419 10.1371/journal.pbio.0020069 PMC368164

[57] Hosokawa T, Koga R, Kikuchi Y, Meng XY, Fukatsu T (2010) Wolbachia as a bacteriocyte-associated nutritional mutualist. Proc Natl Acad Sci U S A 107: 769–774. 20080750 10.1073/pnas.0911476107 PMC2818902

[58] Zientz E, Dandekar T, Gross R (2004) Metabolic interdependence of obligate intracellular bacteria and their insect hosts. Microbiol Mol Biol Rev 68: 745–770. 15590782 10.1128/MMBR.68.4.745-770.2004 PMC539007

[59] Lamelas A, Gosalbes MJ, Manzano-Marin A, Pereto J, Moya A, et al (2011) Serratia symbiotica from the aphid Cinara cedri: a missing link from facultative to obligate insect endosymbiont. PLoS Genet 7: e1002357. 22102823 10.1371/journal.pgen.1002357 PMC3213167

[60] Nakabachi A, Ishikawa H (1999) Provision of riboflavin to the host aphid, Acyrthosiphon pisum, by endosymbiotic bacteria, Buchnera. J Insect Physiol 45: 1–6. 12770389 10.1016/s0022-1910(98)00104-8

[61] Bermingham J, Rabatel A, Calevro F, Vinuelas J, Febvay G, et al (2009) Impact of host developmental age on the transcriptome of the symbiotic bacterium Buchnera aphidicola in the pea aphid (Acyrthosiphon pisum). Appl Environ Microbiol 75: 7294–7297. 19783752 10.1128/AEM.01472-09 PMC2786545

[62] Wu B, Novelli J, Foster J, Vaisvila R, Conway L, et al (2009) The heme biosynthetic pathway of the obligate Wolbachia endosymbiont of Brugia malayi as a potential anti-filarial drug target. PLoS Negl Trop Dis 3: e475. 19597542 10.1371/journal.pntd.0000475 PMC2703803

